# A Delayed Presentation of Perforated Diffuse Large B Cell Lymphoma of the Small Bowel Resulting in a Large Interloop Faecaloma

**DOI:** 10.1155/2022/3881598

**Published:** 2022-05-05

**Authors:** Muhammed A. Zishan, Kasra Raufian

**Affiliations:** Department of General Surgery, Redcliffe Hospital, Anzac Avenue, Redcliffe, Queensland 4020, Australia

## Abstract

Primary small bowel lymphomas are a rare entity but with significant morbidity and a low five-year overall survival even after surgery. Its diagnosis is often delayed due to the lack of clear specific signs, unfamiliarity amongst clinicians, and the lack of screening tools. This often results in patients presenting with tumour-associated complications such as perforation, obstruction, or gastrointestinal bleeding which warrant urgent surgical intervention. We present the case of a patient presenting with a perforated small bowel lymphoma resulting in a large interloop extraluminal faecaloma causing subacute small bowel obstruction. He proceeded to have an emergency open right hemicolectomy and extended small bowel resection to facilitate gross resection of tumour which in conjunction with adjuvant systemic chemotherapy is considered current best practice to manage such neoplasms. Early referral to specialist centres and raising awareness of this rare entity will allow earlier recognition and therefore a more planned approach to the management of such pathology with fewer post-operative complications.

## 1. Introduction

The incidence of primary small bowel lymphoma is on the rise in the western world albeit rare. Previous studies have often treated primary gastrointestinal lymphomas arising from varying sites as a single entity although it is becoming increasingly evident that the optimum treatment regimen and survival rates vary significantly based on site and histology [1]. Given the nonspecific symptoms at presentation, diagnosis is often delayed resulting in emergency presentations with obstruction or perforation. We present here a case of a patient who presented with a subacute small bowel obstruction with an interloop extraluminal faecaloma secondary to a perforated primary small bowel neoplasm which is itself a rare entity accounting for 1-2% of all gastrointestinal tract (GIT) neoplasms [2] Faecalomas are described in literature as a mass of hardened faeces that have failed to pass spontaneously usually within the rectosigmoid and often associated with diseases such as Hirschsprung's, Chagas, inflammatory, psychiatric, and neoplastic processes [3, 4]. This case is unique in terms of the extraluminal location and size of the faecaloma and the underlying aetiology behind this delayed presentation given its rarity which necessitated emergency surgery.

## 2. Case Report

A 62-year-old male presented to the emergency department with a 3-month history of worsening right lower quadrant pain, constitutional symptoms and a CT scan performed in the community revealing a large soft tissue mass measuring 6 cm × 8.5 cm × 7 cm in the right lower quadrant thought to be arising from the distal ileal loops with associated lymphadenopathy. This was on a background of coeliac disease, gastro-oesophageal reflux, and iron deficiency. The only surgical history of note was a right-sided open inguinal hernia repair as a child.

He had recently undergone an endoscopy revealing gastritis and duodenitis and a colonoscopy revealing a 5 mm polyp in the descending colon which was a tubular adenoma with low-grade dysplasia and there was an impression of mass effect at the opening of the terminal ileum; however, no intraluminal sinister lesions were detected. He was awaiting his outpatient appointment with a specialist surgical team regarding his symptoms.

**Figure 1:**
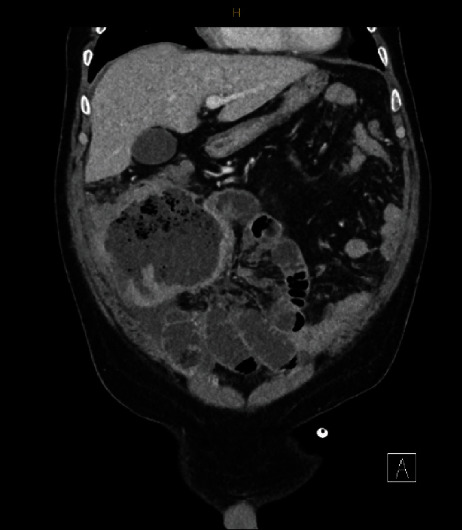
Pre-operative coronal CT images demonstrating the faecaloma.

Given the deterioration of his pain, he presented to the emergency department roughly a few weeks after his colonoscopy. On arrival, he was hemodynamically stable with a low-grade temperature of 37.8°C and tender over his right iliac fossa. Physical examination revealed fullness over his right iliac fossa with a palpable lump over which he was exquisitely tender with focal guarding but without generalised peritonitis. A repeat CT scan was performed as demonstrated in Figure 1, which revealed an interval increase in the size of this complex collection now measuring 10.9 cm × 8.2 cm × 10.6 cm and found to be communicating with loops of small bowel with associated right-sided omental caking and mild ascites consistent with likely underlying neoplasm however differentials included a complex Meckel's diverticulum. A complete blood workup revealed leukocystosis of 21.1 × 10^9^ g/L, thrombocytosis of 600 × 10^9^ g/L, neutrophilia of 14.97 × 10^9^ g/L, lymphocytosis of 4.20 × 10^9^ g/L, and monocytosis of 1.88 × 10^9^ g/L with a normal hemoglobin and the blood film demonstrating toxic changes consistent with an infective process. His electrolytes and liver function tests were grossly normal, with a normal carcinoembryonic antigen (CEA) level. He had an elevated C-reactive peptide of 151 mg/L and was commenced on broad-spectrum IV antibiotics with gut cover and was admitted to the ward for further workup.

**Figure 2:**
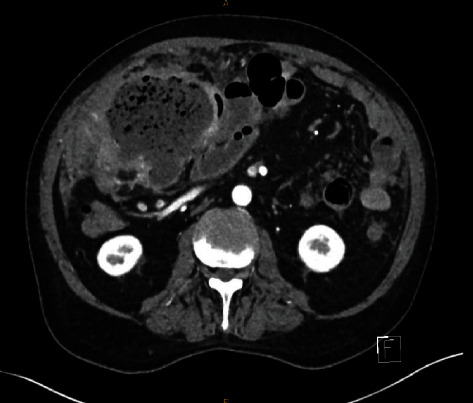
Pre-operative axial CT angiogram revealing relationship of the faecaloma to the ileocolic vessels.

He underwent a CT angiogram of his abdomen and pelvis the following day as depicted on Figure 2 to better characterise this lesion and to assess its vascularity to aid in pre-operative planning. These images revealed findings most consistent with a contained perforation secondary to a neoplastic process (see images). The mass was noted to be adjacent to the ileocolic branches of the SMA without any aberrancy. He continued to have febrile episodes on the ward and a decision was made to undertake exploratory laparotomy on day 2. Given the location and the size of the mass, the local urology team was consulted to facilitate placement of a right-sided ureteric stent which was performed prior to skin incision.

**Figure 3:**
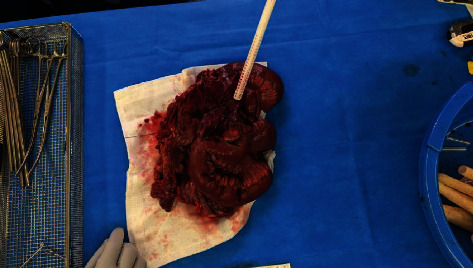
Intra-op specimen revealing site of perforation on evacuating faecaloma contents.

On entering the abdomen via a midline laparotomy, careful adhesiolysis revealed the large 11×11cm collection. Attempts at mobilising this mass resulted in inadvertent spillage of contents which were noted to be enteric in nature. Further abdominal exploration revealed dense fibrotic adhesions to this mass from the terminal ileum to the proximal transverse colon and a few loops of small bowel. An intraopertaive photograph depicts the apperance of this large collection following evacuation of contents in Figure 3. Diffuse mesenteric lymphadenopathy was also noted with multiple peritoneal and omental deposits consistent with likely malignant small bowel perforation. A right hemicolectomy with extended small bowel resection was performed with an ileocolic stapled anastomosis using a GIA 80 mm stapler and two trouser sutures. Rectus sheath catheters were placed for analgesia and a pelvic drain placed. Routine abdominal wall closure with two smaller drains was placed in the subcutaneous tissue and suction dressings over the laparotomy wound were applied to reduce the risk of seroma.

The patient had an uneventful but slow post-op recovery with a gradual upgrade of diet. His drains were removed prior to discharge on day 9 post-op. The histopathology revealed extensive involvement of diffuse large B cell lymphoma not otherwise specified of germinal centre cell type with MYC gene translocation t(8;14). Multiple other deposits of lymphoma were also noted within the small bowel mesentery and omentum. His case was discussed at the local multidisciplinary meeting, and he was subsequently referred onto hematology services where he is currently receiving chemotherapy. He was reviewed in the clinic 3 weeks post-operatively with no concerns, his wounds had healed well, and both the patient and their families were very thankful for the care received.

## 3. Discussion

Given the relative rarity of small bowel lymphomas which make up about 2% of all GIT neoplasms, there is paucity of data to guide its optimal management [2]. Of the primary small bowel neoplasms, 20% of them are secondary to primary lymphomas [1]. It is becoming more apparent that optimal treatment algorithms and survival rates of primary GIT lymphomas vary significantly based on site, with the wide range of histology affecting outcomes [1]. Majority of studies revolving GIT lymphomas have identified that B cell lymphomas are more common than T cell lymphomas with diffuse large B cell lymphoma (DLBCL) being the most common subtype and further subgroup analysis based on site has identified that these tend to have an affinity for the small bowel [5]. Kim et al. have demonstrated in their case series that the ileocecal region is the most affected region within the small bowel as seen in our case [6].

They often present at an advanced stage due to delays in diagnosis as the symptoms may be vague and on most occasions these tumours are found at surgery indicated for other diagnoses or intestinal obstruction as in this case [2]. The most common symptoms of primary intestinal lymphomas include abdominal pain weight loss and anorexia, altered bowel habits, and a palpable abdominal mass as seen in our case [5]. They may present with obstruction or perforation as seen here, with small bowel being the most common site for both and these findings were associated with more aggressive subtypes of B cell lymphoma [1]. Predisposing factors for small bowel malignant tumours include inflammatory bowel disease, GIT polyposis syndromes, and celiac disease [2]. Our patient did have a history of coeliac disease and while coeliac disease is known to be associated with enteropathy associated T cell lymphoma, there is published literature to suggest its association with other Non-Hodgkin B cell lymphomas (NHL) although its pathogenesis is yet to be fully elucidated [7, 8].

Emergency surgery for perforated NHL of the small bowel can be challenging as the underlying pathology is often diagnosed at the time of operation [5]. In this case, while the CT findings were highly suggestive of a malignant process, it was only confirmed intra-operatively. Small bowel lymphomas are usually treated by resection with or without anastomosis based on the clinical picture with post-operative sepsis being the major complication in these cases [9]. There is paucity of high-quality evidence to guide management in emergency treatment of NHL of the small bowel given the rarity of this condition and the ever-evolving histological classifications making it difficult to draw conclusions from published literature.

A recent retrospective study published in 2021 from a national database in the United States found that adjuvant chemotherapy following surgical resection is associated with an improved overall survival in primary small bowel lymphoma as opposed to surgery alone; however, they did exclude patients with stage IV disease and included both elective and emergency presentations making it difficult to apply to our case; however, the patient in our case did proceed to receiving chemotherapy [1]. An earlier, smaller, single centre retrospective study in 2017 also found that a combined chemotherapy and surgery approach with gross resection of tumour defined as intraoperative removal of tumour macroscopically was associated with improved overall 5-year survival [10]. They also noted that emergency surgery did not lead to poorer prognosis and that the risks of early mortality or post-operative complications were identical to an elective setting [10]. These findings help solidify the role of surgery in the management of small bowel lymphoma in both emergency and elective setting and dispel the notion of delaying surgery until tumour-related complications arise to avoid postponement of chemotherapy in the event of developing complications post-operatively [10]. The patient in our case had a very satisfactory post-operative outcome and proceeded straight to chemotherapy within a month of being discharged from the hospital.

In conclusion, primary care clinicians need to be aware of the red flag symptoms in the community that warrant urgent referral to specialists to avoid delayed presentations with tumour-associated complications which would allow elective surgical management in a more controlled fashion with fewer post-operative complications, e.g., anastomotic leaks and wound infection, favour bowel preservation and early review by oncologists to facilitate timely healthcare delivery and a better patient experience.

## Data Availability

The data used to support the findings of this study are included within the article.
